# Posttransplant Lymphoproliferative Disease after Pediatric Solid Organ Transplantation

**DOI:** 10.1155/2013/814973

**Published:** 2013-09-24

**Authors:** Martin Mynarek, Tilmann Schober, Uta Behrends, Britta Maecker-Kolhoff

**Affiliations:** ^1^Department of Pediatric Hematology and Oncology, Hannover Medical School, 30625 Hannover, Germany; ^2^Integrated Research and Treatment Center Transplantation, Hannover Medical School, 30625 Hannover, Germany; ^3^Dr. von Haunersches Kinderspital, Ludwig-Maximilians-University Munich, 80337 Munich, Germany; ^4^Clinical Cooperation Group Pediatric Tumorimmunology, Children's Hospital, University of Technology Munich, Helmholtz Center Munich, 80804 Munich, Germany

## Abstract

Patients after solid organ transplantation (SOT) carry a substantially increased risk to develop malignant lymphomas. This is in part due to the immunosuppression required to maintain the function of the organ graft. Depending on the transplanted organ, up to 15% of pediatric transplant recipients acquire posttransplant lymphoproliferative disease (PTLD), and eventually 20% of those succumb to the disease. Early diagnosis of PTLD is often hampered by the unspecific symptoms and the difficult differential diagnosis, which includes atypical infections as well as graft rejection. Treatment of PTLD is limited by the high vulnerability towards antineoplastic chemotherapy in transplanted children. However, new treatment strategies and especially the introduction of the monoclonal anti-CD20 antibody rituximab have dramatically improved outcomes of PTLD. This review discusses risk factors for the development of PTLD in children, summarizes current approaches to therapy, and gives an outlook on developing new treatment modalities like targeted therapy with virus-specific T cells. Finally, monitoring strategies are evaluated.

## 1. Introduction

Progress in solid organ transplantation (SOT) dramatically improved the prognosis for children and adolescents with hereditary or acquired terminal organ failure. Immunosuppressive induction and maintenance regimens were instituted to prevent organ graft rejection by the recipient's immune system. On the downside of pharmacological immunosuppression, a decreased immunological surveillance of infections and malignancies is observed. Pediatric and adolescent patients after SOT carry an increased risk of cancer development, which is estimated to exceed the normal population's up to 45-fold, depending on the type of cancer [1]. The most frequent malignant complications in children are posttransplant lymphoproliferative diseases (PTLDs), often arising in the context of prior Epstein-Barr virus (EBV) infection. The incidence of PTLD depends on the type of organ transplanted, the respective intensity of immunosuppression, and the recipient's viral status prior to transplantation; it varies between 1 and 2% in pediatric renal transplant recipients and up to 20% in recipients of lung or intestinal transplants [2–4].

This review focuses on special characteristics of pathogenesis, treatment, and prognosis of PTLD in children and adolescents after SOT.

## 2. Pathophysiology

Pathophysiology of PTLD is only partially understood, and its etiology is most probably multicausal. Despite all uncertainties, EBV infections and transplant-related immunosuppression are unquestioned elements of posttransplant lymphomagenesis.

### 2.1. EBV Infection

EBV is a human oncovirus belonging to the group of gammaherpesviruses. Primary infection with EBV usually occurs during childhood or adolescence, and by the age of 30, more than 90% of the population have become seropositive [5]. 

Directly after B-cell infection, EBV establishes a nonproductive (“latent”) infection that is divided into four types (latency type 0 to 3) characterized by distinct viral gene expression profiles [6]. Upon specific stimulation, EBV may switch into a productive (“lytic”) mode of infection, in which viral progeny is produced by the infected cell.

### 2.2. EBV-Driven B-Cell Proliferation

*In vitro* EBV infection of B cells results in the outgrowth of immortalized lymphoblastoid B-cell lines (LCLs), which express the latency type 3 program. This “growth program” is characterized by the expression of nine proteins: three latent membrane proteins (LMPs) and six EBV-associated nuclear antigens (EBNAs). These mimic external growth signals (LMP1 and LMP2) or directly regulate gene expression (EBNA2, EBNA3c), thereby driving the infected cell into proliferation [7]. 

In type 2 latency (“default program”), EBV gene expression is limited to the LMPs and EBNA1. Hereby, EBV supplies the infected B-cell with signals, which are usually received upon antigen contact in the germinal center. These signals drive the infected cell towards the memory B-cell stage. In type 1 latency, only EBNA1, a gene required to maintain the viral genome during mitosis, is expressed. In latency type 0, no EBV protein is expressed in the infected cell [8, 9].

Induction of lytic replication in some of the latently infected cells leads to the production and release of infectious viral progeny that can infect neighboring B cells, thereby promoting virus spreading and EBV-associated B-cell proliferation [8].

The contribution of EBV to the etiology of PTLD is inferred by the high proportion of EBV-positive pediatric PTLDs (70%) [3, 10], which is much higher than that observed within the B-cell reservoir of latently infected healthy EBV carriers, where only one in 1,000 to 100,000 peripheral B cells is EBV-positive [11]. 

### 2.3. Impaired T-Cell Control of EBV-Induced B-Cell Proliferation

EBV-infected B cells normally induce strong CD8+ and CD4+ T-cell responses, which control the proliferation of infected B cells *in vitro* and *in vivo*. The T-cell response is directed against a broad set of viral gene products expressed either during the latent or the lytic reactivation cycle of EBV and, in addition, against some cellular antigens that might either mimic viral antigens or are overexpressed in the context of EBV infection [12].

For establishing LCL from peripheral blood *in vitro*, this potent T-cell response has to be inhibited, either by using immunosuppressants like cyclosporine A (CSA) or by removing T cells from the cell population.

Impaired T-cell responses *in vivo* due to primary or secondary immunodeficiency greatly increase the risk of uncontrolled B-cell proliferation. For example, transplant recipients receiving medical immunosuppression as well as patients suffering from the acquired immunodeficiency syndrome (AIDS) carry an elevated risk of EBV-associated lymphomas [8].

### 2.4. Additional Factors

Despite its undoubted role, EBV infection alone may not be sufficient to induce PTLD [13, 14]. Some characteristic mutations (e.g., c-myc translocations in Burkitt's or Burkitt-like PTLD [15]) have been described. How these genetic alterations collaborate with EBV in B-cell transformation and lymphomagenesis remains unknown. 

## 3. Clinical Risk Factors for Development of PTLD

### 3.1. EBV

EBV seronegativity at transplantation is a very potent risk factor for the development of PTLD. Seronegative patients carry a 4-fold increased risk of PTLD [16–19]. The prognostic value of EBV monitoring after transplantation will be discussed separately.

### 3.2. CMV

Like EBV, seronegativity for the cytomegalovirus (CMV) at the time of transplantation is a potential risk factor for PTLD, although the effect is not as strong as for EBV and less consistent [19, 20]. 

### 3.3. Age at Transplantation

Organ recipients younger than 18 years at transplantation are believed to have a 2- to 4-fold higher risk to develop PTLD than adult transplant patients (Table 1) [17, 21]. Within the pediatric age group, there is some evidence of an increased risk in younger children [16, 22], although this is not consistent between studies [23]. EBV has been suggested to be the link between age and PTLD risk. Younger children are more likely to be EBV-negative at transplantation and therefore at higher risk for PTLD development.

### 3.4. Organ Graft

A large recent study on PTLD in both pediatric and adult SOT recipients with more than 160,000 participants revealed an approximately doubled PTLD incidence in liver transplant (LTx) recipients compared to renal transplant (RTx) recipients (Table 1). The same was true for the comparison of RTx recipients to heart transplant (HTx) recipients (hazard ratio 1.2 for EBV-negative recipients, *P* = 0.222) [17].

Among pediatric patients, overall incidences for PTLD are smallest in RTx recipients with 2-3% at 5 years posttransplantation [21, 22] and 6% in pediatric HTx recipients 5 years after transplantation [16]. Data on PTLD incidence after pediatric LTx are available from small case series, usually from single centers [24–26]. Herein, overall incidences of 5–10% are reported, but median followup for the entire cohort was either longer than 5 years [24, 25] or not reported [26]. PTLD is most frequent in pediatric lung transplant recipients (overall PTLD incidences of approximately 15% [27, 28]) or recipients of intestinal grafts (predicted incidence 20 ± 5%) [29].

Mismatches in the HLA-DR locus between graft and recipients seem to be important at least in kidney transplant recipients: in a large retrospective analysis a complete HLA-DR mismatch confers a 2-fold increased risk of PTLD development [30]. 

### 3.5. Time Point after Transplantation

**Figure 1:**
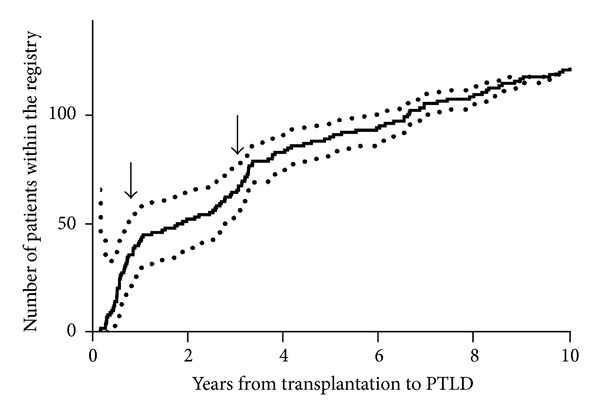
Time from transplantation to diagnosis of PTLD of 127 patients in the German Ped-PTLD registry. Kaplan-Meyer curve (continuous line) and 95% confidence intervals (dotted curve). Note the rapid increase within the first year and another in the third year.

Time from transplantation to PTLD has a bimodal distribution in children with one peak in the first year after transplantation (early PTLD) and another in the second to third year (Figure 1) [10, 15]. Almost all early PTLDs are EBV-associated and frequently present atypically with extranodal or graft organ involvement. Late PTLDs are more likely to present as “classical” lymphoma and often display the diagnostic oncogenic mutations of their counterparts in immunocompetent patients (i.e., translocations involving the c-myc locus) [10].

### 3.6. Type of Immunosuppression

#### 3.6.1. Induction with Monoclonal Antibodies

T-cell depleting antibodies like antithymocyte globulin (ATG) or muromonab-CD3 (OKT3) as well as interleukin-2 (IL-2) receptor specific antibodies like basiliximab or daclizumab are widely used to induce graft tolerance, but their impact on PTLD development is difficult to estimate [31]. Best evidence is available for OKT3 to increase PTLD rates [22, 32–34]. Still, in some studies low incidences of PTLD even in the context of T-cell depleting antibodies have been observed [35] suggesting that confounding factors might play an important role.

#### 3.6.2. Immunosuppressive Maintenance

Although the influence of immunosuppression in the pathogenesis of PTLD is widely accepted, there is no consensus about the impact of a single agent. Differences between CSA and tacrolimus have been discussed in several studies [17, 22, 36]. In some studies a higher risk of PTLD has been associated with the use of tacrolimus, while others were unable to find significant differences. However, most data derive from retrospective studies, they still need to be confirmed in prospective controlled trials. 

Mycophenolate mofetil (MMF) is virtually always used in combination with other immunosuppressive drugs, hampering the possibility to analyze its influence separately. Despite these limitations, the addition of MMF does not seem to increase the risk of PTLD [18, 37, 38]. In contrary, some studies suggest that the risk may be reduced in selected patients [37, 38], but the results are too inconsistent to draw final conclusions.

Protective effects were proposed for immunosuppressive drugs belonging to the group of mammalian target of rapamycin inhibitors (mTOR-I), because mTOR signaling pathways are activated in many PTLD cases [39, 40]. Nevertheless, recent studies reported slightly increased PTLD incidences during maintenance therapy with mTOR-I-based immunosuppression [19, 41]. 

## 4. Classification

Since symptoms of PTLD are often unspecific, diagnosis of PTLD requires cell and/or tissue sampling for histopathologic examination. Per definition, every lymphoid malignancy arising after transplantation is classified as PTLD. The histological characteristics are not specific, therefore knowledge about the transplant history is essential for the pathologist to differentiate PTLD from other types of lymphoproliferation. The 2008 WHO classification for lymphoid malignancies [42] divides PTLD into four major categories: early lesions, polymorphic PTLD, monomorphic PTLD, and Hodgkin's disease/Hodgkin-like PTLD. These are often difficult to differentiate, and an experienced pathologist is required to make the definitive diagnosis. Especially in pediatric patients, an external review of PTLD-suspected tissue samples is highly recommended. 

### 4.1. Early Lesions

Early lesions show oligo- or polyclonal proliferations of EBV-positive B cells while the underlying tissue architecture is preserved. The B cells may either have a predominantly immunoblastic phenotype (infectious mononucleosis like early lesion PTLD) or a plasma-cell phenotype (plasmacytic hyperplasia early lesion PTLD). 

### 4.2. Polymorphic PTLD

Like early lesions, polymorphic PTLDs demonstrate oligo- or polyclonal B-cell proliferations, but here, the infiltrating cells destroy the original architecture of the host tissue. While polymorphic PTLD can easily be differentiated from early lesions in lymph nodes, this can be very difficult in extranodal PTLD [43].

### 4.3. Monomorphic PTLD

**Figure 2:**
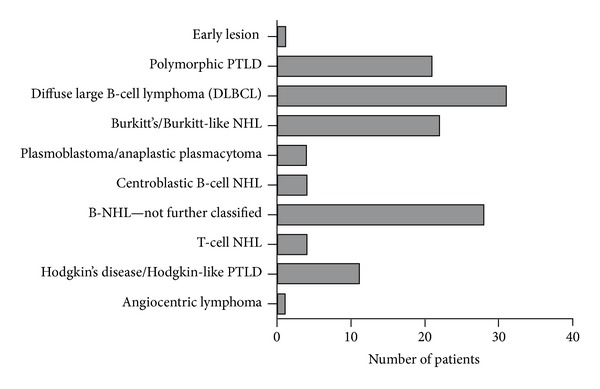
Distribution of histological subtypes of pediatric PTLD reported to the German Ped-PTLD registry.

All PTLDs fulfilling the histopathologic criteria of “classical” non-Hodgkin's lymphoma (NHL) are diagnosed according to the classification of nontransplant associated lymphomas. Within pediatric registry studies, monomorphic PTLDs account for 35–83% of all cases [3, 10, 15]. The high variability may be in part explained by underreporting of early stage PTLD that resolves with reduction of immunosuppression alone. B-cell lymphomas (in particular diffuse large B-cell lymphoma, (DLBCL)) represent the most frequent single entity (Figure 2). T-cell NHL or plasmacytoma is less frequently observed. Monomorphic PTLD may be monoclonal or polyclonal by molecular IgH or T-cell receptor analysis. Some more specific entities are characterized by chromosomal translocations, for example, Burkitt's lymphoma by the rearrangement of the c-myc locus.

### 4.4. Hodgkin's Disease/Hodgkin-Like PTLD

Classical Hodgkin's disease and Hodgkin-like PTLD also belong to monomorphic PTLDs, but due to their special histological and clinical features, they represent a separate group within the WHO classification. 

## 5. Therapy

The two basic principles of current standard PTLD treatments are reconstitution of anti-EBV/antitumor immune responses, and, if this is not sufficient, antineoplastic immuno-/chemotherapy (+/− radiotherapy). Both approaches are limited by the patients' pre-existing condition: reconstitution of immune responses carries the risk of transplant rejection, which may be intolerable in some circumstances. On the other hand, the patients may be unable to tolerate the side effects of chemotherapy or radiotherapy due to their medical history.

### 5.1. Reconstitution of the Immune Response

Reduction of immunosuppressive drugs (RIS) is a key component of PTLD therapy in every patient. It may be sufficient to induce complete remission (CR) as a single therapeutic strategy in some patients [44]. However, it has to be taken into account that transplant rejections occur in about 40% of all RIS patients [45, 46] and require re-intensification of the immunosuppressive regimen. Balancing immune reconstitution versus the risk of graft rejection is therefore challenging and requires expert guidance and interdisciplinary cooperation of transplant physicians and oncologists. 

Current guidelines recommend RIS wherever possible, but due to heterogenous strategies, the documented effectiveness of RIS still varies between studies. While some retrospective studies report CR in up to 50% of the patients [46], the only available clinical trial that included systematic RIS did not demonstrate any CR and achieved partial remissions (PR) in only 1/16 patients [45]. However, a selection bias may have led to an underestimation of the effectiveness of RIS in this trial, because histological confirmation was mandatory for all patients. This might have led to the inclusion of patients with poor risk factors responding poorly to RIS. In children, the effectiveness of RIS is even less clear. Anecdotal data report survival rates of 30–73% with RIS alone [36, 47–49].

However, when RIS is given to PTLD patients, very close monitoring is mandatory to recognize progressive disease and/or rejection as early as possible [50, 51]. In adult patients, elevated lactate dehydrogenase (LDH), organ dysfunction at diagnosis or multiorgan involvement of PTLD [46] may help identify patients at high risk of RIS treatment failure who might require further therapy [51]. No parameters are published to predict the response to RIS in pediatric patients. Therefore, patients with advanced disease, patients in poor clinical conditions, and patients with rapid disease progression should be considered as high risk patients who might need supplementary or alternative treatment. 

### 5.2. mTOR Inhibitors

mTOR-inhibitors like rapamycin/sirolimus and everolimus are currently evaluated in small clinical trials for their therapeutic effect in hematologic malignancies, and some promising data have been published [52]. mTOR signaling pathways have been found activated in PTLD tissue [39], and antiproliferative effects of rapamycin on EBV-transformed B-cell lines have been observed *in vitro* [53]. *In vivo*, encouraging response after a conversion from calcineurin inhibitors to mTOR-I has been reported [54]. A detailed analysis of the interplay between EBV, PTLD, and mTOR-I is given in [55]. Therefore, a change in immunosuppressive therapy towards an mTOR-I-based regimen may be beneficial, although this rationale still needs to be evaluated in prospective trials. It is an interesting notion that these drugs seem to be beneficial for the treatment of PTLD, while their impact in PTLD prevention/prophylaxis may be disadvantageous (see Section 3.6.2).

### 5.3. Antineoplastic Drugs

Often, modification of immunosuppression will not be sufficient to induce long-term complete remission of PTLD. Cytoreductive drugs (antibodies and/or chemotherapy) are necessary to substantially control PTLD activity in most patients. 

#### 5.3.1. Rituximab

After the inclusion of the anti-CD20 antibody rituximab into standard regimens, outcome of PTLD treatment improved drastically [56]. A 2-year progression-free survival of >40% was achieved with rituximab monotherapy in adults [57–59], but additional cytotoxic chemotherapy was required to cure PTLD in the majority of adult patients [60]. 

After documentation of favorable responses to rituximab also in pediatric PTLD [61, 62], rituximab has become standard element of the treatment for CD20 positive pediatric PTLD [63, 64]. The German Pediatric PTLD study group developed a phase II clinical trial protocol (“Ped-PTLD 2005-Pilot”) to test a sequential stratified treatment strategy of rituximab monotherapy and moderate chemotherapy in children with PTLD after SOT. After three weekly doses of rituximab at 375 mg/m², children were stratified according to their responses: patients with PR or CR continued with rituximab monotherapy for another three doses every three weeks, while nonresponders received chemotherapy. The data analysis is currently ongoing.

#### 5.3.2. Cytotoxic Drugs

Anthracycline-based chemotherapy (e.g., cyclophosphamide, hydroxydaunorubicin, oncovin/vincristine, prednisone (CHOP)) in combination with rituximab is the standard of care for adult patients with PTLD that are able to tolerate the toxicity associated with this regimen [51, 65, 66]. Other anthracycline-based regimens have also been used successfully in PTLD patients [67]. Very promising results have recently been reported after sequential treatment of four weekly courses of rituximab followed by four courses of CHOP-21 [60]. 

In children, a recent prospective phase II clinical trial on treatment of PTLD combined rituximab (375 mg/m² weekly for six consecutive weeks) with simultaneous low-dose chemotherapy (six three-weekly courses of cyclophosphamide 600 mg/m² at day 1 together with prednisone 1 mg/kg/d day 1–5) [63]. Hereby, 69% of all patients achieved CR. The estimated 2-year overall survival (OS) was 83%. 

Patients who do not respond to low-dose chemotherapy or relapse require second-line chemotherapy. Data on safety and efficacy related to the suggested regimens are scarce. The German pediatric PTLD study group recommends intensive chemotherapy according to *de novo* NHL protocols in case of primary treatment failure (e.g., NHL-BFM protocols [68]). 

Allogeneic hematopoietic stem cell transplantation has been reported as a salvage therapy for PTLD in few case reports [69], but the available results were disappointing. 

### 5.4. Radiation Therapy

There is very limited data on the significance of radiation therapy in the treatment of pediatric PTLD. In first-line treatment, involved field radiation in Hodgkin's/Hodgkin-like PTLD and cranial irradiation for CNS-PTLD may represent curative treatment elements. However, radiotherapy in pediatric PTLD patients is usually considered only in second-line treatment, both as part of salvage concepts and in palliative care situations. 

### 5.5. Treatment for Rare PTLD Types

#### 5.5.1. Burkitt's or Burkitt-Like PTLD

Burkitt's or Burkitt-like PTLD is more aggressive compared to “classical” DLBCL-type PTLD [15, 70, 71]. Adult patients seem to respond well to standard treatment in some reports [71], while in others more aggressive therapy is recommended [51]. In pediatric patients, rituximab monotherapy is probably insufficient [70]. However, the intensity of chemotherapy regimens is still under discussion and has to be determined in ongoing and future prospective studies. 

#### 5.5.2. T-Cell PTLD

T-cell PTLD is a rare entity [72], especially in the pediatric population. Approximately 20 pediatric T-cell PTLDs have been reported to date [72, 73]. T-cell PTLD requires polychemotherapy to induce remission [73], but the prognosis still remains poor.

#### 5.5.3. Hodgkin or Hodgkin-Like PTLD

Hodgkin or Hodkgin-like PTLD accounts for approximately 3–9% of pediatric PTLDs [10, 15] and 9% of adult PTLD [74]. Therapy recommendations are based on individual case reports. Most authors recommend treatment according to the therapy guidelines of *de novo* Hodgkin's lymphoma. 

#### 5.5.4. PTLD with CNS Involvement

Treatment for PTLD affecting the central nervous system (CNS) is difficult, because most drugs used in standard therapy (especially rituximab) do not sufficiently cross the blood-brain barrier. Therapeutic attempts with both high-dose intravenous rituximab [75] and intrathecal rituximab administration [76, 77] have been made to deliver rituximab into the CNS. Moreover, both CNS irradiation [78] and high-dose methotrexate have been administered [79], but the most efficient approach remains to be determined.

### 5.6. Nonstandard/Experimental Therapy Elements

#### 5.6.1. Antiviral Drugs

Assuming that active EBV replication contributes to the pathogenesis of EBV-positive PTLD, ganciclovir [80] or cidofovir [81] have been used for the treatment of PTLD. However, effectiveness has not been shown, and a direct effect on latently EBV infected tumor cells is unlikely, because these cells do not express the viral protein kinase that is essential for the drug's activity [82]. Thus, there is little rationale for antiviral treatment of overt PTLD.

To sensitize EBV-positive PTLD to antiviral drugs, experimental approaches aim at inducing lytic replication of EBV in latently infected cells. Within the course of the lytic cycle, the viral kinase is expressed, which can induce ganciclovir's cytotoxic activity. A clinical phase I/II trial using ganciclovir together with arginine butyrate as inductor of lytic replication activity resulted in CR in a reasonable number of patients with refractory EBV-positive lymphoma [83].

Anecdotal responses to antiviral treatment with foscarnet, a drug that does not require activation by viral kinases, have been reported in adult patients [84]. The significance of foscarnet and other antivirals (e.g., cidofovir) in the treatment of pediatric PTLD remains mainly elusive. 

#### 5.6.2. EBV-Specific T Cells

EBV proteins expressed in a high proportion of pediatric PTLD tumors are potential targets for tumor-specific immunotherapy via adoptive transfer of virus-specific cytotoxic T-lymphocytes (EBV-CTLs). An overview of published clinical trials is given in Table 2. Promising results in SOT patients have been obtained with either autologous, *ex vivo* expanded EBV-CTLs [85–88] or EBV-CTLs derived from healthy, partially HLA-matched third-party donors [89, 90]. With third-party EBV-CTLs, response rates were approximately 50% in patients with PTLD after SOT, who had failed to respond to at least one prior treatment. Although third-party EBV-CTLs in SOT patients were only partially HLA-matched, there was no evidence of EBV-CTL-related graft-versus-host disease (GvHD), and no other significant toxicities were reported.

Unfortunately, the production of *ex vivo* expanded EBV-CTLs for clinical use by repetitive antigenic stimulation is very laborious and expensive and therefore performed in only few institutions [91]. The process requires several weeks of in vitro cell culture. To offer EBV-CTLs when clinical need is urgent, banking of EBV-CTLs was suggested [92]. 

Availability of EBV-CTL is not only limited by labour intensity and costs but also by the fact that the production has to follow the standards of good manufacturing practice (GMP) for open cell culture processes. Therefore, more rapid and easier strategies for the generation of EBV-CTLs are currently being developed. They aim at direct isolation of EBV-CTLs from donor-derived peripheral blood mononuclear cells (PBMC) by labeling with magnetic beads and subsequent purification via magnetic columns. Specific labeling of EBV-CTL is either achieved by the use of EBV-epitope major histocompatibility complex (MHC) class I multimeres [93] or by cytokine secretion and capture after stimulation with EBV-derived antigen [94, 95]. As recently reviewed by Pagliara and Savoldo [96], both strategies may allow for a fast and relatively easy generation of T-cell products and thereby increasing the products' availability. However, they have important limitations. First, the spectrum of known EBV epitopes is still limited, and selected epitopes might not sufficiently cover the whole EBV antigenic repertoire expressed by the individual PTLD tumor cells. To overcome these limitations, the inclusion of virus-like particles into future stimulation protocols was suggested [97]. Second, until recently, EBV epitope-specific MHC class I multimers allowed for the isolation of EBV-specific CD8+ but not MHC class II-restricted CD4+ T cells. Thus important cytotoxic and helper T-cell subpopulations may have been missed. With the recent introduction of EBV epitope-specific MHC class II multimers, EBV-specific T-cell preparations may contain a more comprehensive set of specificities and cell populations in the future [98]. For a more detailed review of EBV-CTL therapy, see Bollard et al. [99].

### 5.7. Prognosis

The prognosis of PTLD in pediatric patients with PTLD is better than in adult patients. In two prospective studies the 2-year overall survival was 73% in the prerituximab era [100] and 83% in the rituximab-complemented trial [63]. In retrospective series, 2-year overall survival of unselected PTLD patients was around 70–80% [3, 10, 15]. No significant prognostic factors were identified in the prospective trials by Gross and colleagues. In retrospective analyses bone marrow or CNS involvement, EBV-negative tumors, lack of CD20 expression and very early or late PTLD development were adverse prognostic factors in terms of survival ([15, 23, 101, 102] and BMK, unpublished results). However, historical data need to be confirmed in prospective clinical trials, especially regarding the impact of rituximab introduction in the early new millennium.

## 6. Monitoring/Prevention of PTLD

### 6.1. Monitoring

Several efforts have been taken to define parameters estimating the risk of PTLD development and warranting preemptive measures. 

#### 6.1.1. EBV DNA Load

Serial monitoring of EBV DNA load in the peripheral blood by polymerase chain reaction (PCR) has been proposed as a diagnostic tool to identify patients at high risk of PTLD development. Both the magnitude and the duration of EBV detection in peripheral blood were suggested as critical parameters in PTLD risk estimation. Data from several retrospective analyses point towards a heterogenic picture: pediatric HTx patients with chronic high load (CHL) seemed to be at an increased risk for PTLD development [103, 104], while most LTx patients with CHL did not develop PTLD [105]. The role of CHL in intestinal transplant recipients is inconclusive [106]. A recent large prospective study did not reveal any predictive value of very high or sustained EBV DNA in the peripheral blood of 106 pediatric RTx patients; remarkably, all three PTLD patients in this study had documented EBV infection or reactivation prior to PTLD development [13]. 

Different methods of EBV load monitoring are currently used in different laboratories, and the lack of standardization makes it difficult to compare results between centers and draw specific conclusions from individual cut-off values. There seems to be a difference between EBV DNA load in serum and whole blood samples. Stevens et al. demonstrated that early PTLD detection can be achieved in Lung-Tx patients by measuring EBV DNA in the cellular blood compartment, while parallel serum samples were all below the cut-off value [107], most likely due to latent EBV infection of circulating B cells without significant virus production and release into body fluids. However, questions remain about the optimal amplified region of the EBV genome and the best-suited specimen for EBV DNA detection depending on the EBV-associated disease of interest. For a more detailed review on current best practice and future requirements for determining EBV load, see Ruf and Wagner [108].

#### 6.1.2. EBV-Specific T-Cell Response

Monitoring of cellular immune responses to EBV in addition to EBV DNA load in peripheral blood was suggested to identify patients at risk for PTLD early after transplantation [109]. In our own analysis, EBV-specific T cells appeared to be reduced in early but not late PTLD patients [110]. Earlier studies had suggested that EBV-specific CD4+ T cells have significant diagnostic impact. The absolute CD4+ T-cell count was lower in PTLD patients [111], and higher numbers of CD4+ T cells in infused CTL lines were associated with better responses of PTLD to T-cell therapy [89]. However, due to the very low levels of EBV-specific CD4+ T cells in peripheral blood, their rapid detection in transplant patients remains challenging. 

#### 6.1.3. Serological Parameters

Several serological markers have been evaluated as surrogate markers to predict PTLD development. EBV induces the expression of CD30 on target cells, and soluble CD30 (sCD30) secreted into the serum has been found elevated in patients with PTLD [112]; however, high levels were also detected in patients with primary EBV infection and thus were not specific for PTLD. Whether plasma markers of B-cell dysfunction might help identify recipients at high risk of PTLD is still under investigation [113]. Increased levels of inflammatory proteins like IL-6 or IL-10 were documented in PTLD patients [114], but they were either unspecific (IL-6) or did not correlate with the course of the disease (IL-10). Our group has recently identified CXCL13, a homeostatic B-cell chemokine, to be elevated in serum of patients with PTLD [115]. In anecdotal cases elevation of CXCL13 preceded the development of PTLD by several months; however, the sensitivity and specificity of serum CXCL13 as high PTLD risk marker needs to be confirmed in a prospective patient cohort.

#### 6.1.4. Monitoring of Patients during and after Treatment for PTLD

For monitoring of response to PTLD treatment, clinical and radiologic examinations (MRI, ultrasound) still represent the gold standard. Techniques quantifying metabolic activity of morphologic lesions (e.g., ^18^FDG-PET scan) may prove useful to individually tailor treatment in patients with residual lesions [116] similar to developing strategies in nonimmunocompromised individuals. While EBV load monitoring did not correlate with treatment response in a small adult series, longitudinal monitoring of serological markers like IL-6 [114] or CXCL13 [115] may allow for early identification of treatment failures. 

In patients after successful treatment of PTLD monitoring should be performed as suggested by guidelines for lymphomas in nonimmunocompromised persons. The followup is based on regular clinical and radiologic evaluation of remission status. Redetection of EBV in peripheral blood of patients treated with rituximab does not seem to predict disease relapse but may rather be a surrogate of normal B-cell recovery [110, 117]. Whether serological and/or immunological markers may prove useful to detect disease recurrence is subject of ongoing clinical trials.

### 6.2. Prevention of PTLD

The ultimate goal will be to completely abrogate PTLD development by effective preventive measures. Some measures have been suggested in high-risk patients, but all require further confirmation until they can be considered standard of care in SOT recipients.

#### 6.2.1. CMV Immunoglobulin

Opelz and colleagues analyzed the effect of CMV immunoglobulin and antiviral drugs in more than 42,000 renal transplant recipients [118]. None of the patients who received CMV immunoglobulin for CMV prophylaxis developed PTLD during the first posttransplant year arguing for a possible early preventive effect in high-risk patients. The prophylaxis had no impact on the development of late PTLD. However, chemoprophylaxis with ganciclovir, valganciclovir, or acyclovir was associated with a reduction in the risk of early PTLD [18] and a significantly lower incidence of EBV primary infection in pediatric RTx patients [13]. Antiviral drugs are therefore used for PTLD prophylaxis by many SOT centers early after Tx in high risk patients. 

#### 6.2.2. EBV-Specific T Cells

Adoptively transferred EBV-specific T cells were not only shown to have therapeutic effects in manifest PTLD but also to prevent PTLD when given to high risk patients prophylactically after stem cell transplantation [91] or SOT [119]. However, the limitations of adoptive T-cell transfer for PTLD prophylaxis are the same as for PTLD therapy. For a more detailed review of clinical results on therapeutic and preemptive strategies using EBV-specific T cells, see [120].

#### 6.2.3. Immunization against EBV

Strategies of active immunization against EBV prior to SOT have been advocated to avoid subsequent EBV primary infection, latent infection, and putative cancer development. In a phase II clinical trial a recombinant DNA vaccine targeting the EBV lytic cycle glycoprotein gp350 reduced the incidence of infectious mononucleosis by 78% in healthy young adults [121]. However, asymptomatic infection with EBV was not reduced in the vaccinated group, and the effect on prevention of cancer development remains to be determined. A small phase I clinical trial evaluated the immunogenicity of a dose-reduced gp350 vaccine in children on dialysis awaiting renal transplantation. The results were disappointing with respect to the induction of neutralizing antibodies [122]. As reviewed in [123], current strategies for EBV vaccine development are focusing on the induction of protective T-cell responses rather than neutralizing antibodies to prevent EBV-associated diseases including EBV-associated cancer.

## 7. Perspectives

Since the first description of PTLD in 1969 [124], considerable improvement in diagnosis, treatment, and understanding in both adult and pediatric SOT patients has been achieved. However, PTLD still represents a major threat to SOT recipients accounting for significant posttransplant morbidity and mortality. Moreover, prognosis is still poor, and future research is urgently needed. Hereby, two important points may be considered.

At first, many previous diagnostic and therapeutic attempts have been hampered by the pronounced heterogeneity and relative rarity of the disease. We increasingly understand that there are actually several separate disease entities summarized under the diagnosis of “PTLD.” A better definition of these entities and tailoring of specific treatment schemes are warranted. This can only be achieved by multicentre and international transplant and/or PTLD registries and research collaborations. Ongoing or recently published prospective trials such as Ped-PTLD or the study by Children's Oncology Group [63] are important steps in that direction.

Secondly, new insights into cellular and molecular mechanism need to be better incorporated into clinical research projects. So far, primary diagnostics, risk assessment, and response evaluation mainly rely on clinical parameters. New approaches such as sequencing studies and gene expression profiles [132] should help define distinct disease entities and may provide rationales for new therapeutic targets. For EBV-positive PTLD, the inclusion of EBV specific T cells into standard therapy regimens most certainly provides a promising step towards a more specific antineoplastic therapy. Efforts are needed to optimize the production of CTL for adoptive transfer and make this treatment available for more patients.

## Figures and Tables

**Table 1:** Pediatric and adult PTLD.

	Pediatric PTLD		Adult PTLD	
		Reference		Reference
EBV serostatus at transplantation				
Negative	78%	[10]	13%*	[125]
Positive	22%		87%*	
EBV association of PTLD	90%	[10]	45% (−69%*)	[60, 125]
Histology				
Polymorphic	16%	[10]	27%*	[125]
Monomorphic	72%		65%*	
Hodgkin's disease	9%		7%*	
B-cell origin	97%		93%*	
T-cell origin	3%		6%*	
First-line treatment (B-cell disease)	Rituximab +/− reduced intensity chemotherapy	[63]	Rituximab +/− standard chemotherapy	[60]
Prognosis	2-year overall survival 80–90%	[63]	2-year overall survival 60–70%	[60]
Incidence according to transplanted organ				
Kidney	2%-3%	[21, 22]	1.0%–2.3%	[4]
Liver	5%–10%	[24–26]	1.0%–2.3%	
Heart	~6%	[16]	1.0%–6.3%	
Lung	~15%	[27, 28]	2.4%–10.0%	
Small bowl	~20%	[29]	20%	

*Adult data derived from a KTx population.

**Table 2:** Selected studies on the treatment of EBV infection or EBV-related posttransplant lymphoproliferation with adoptively transferred EBV-specific T cells.

Authors	Number of patients	Indication for EBV-CTL	EBV-CTL donor	EBV-CTL preparation*	Number of doses	Total number EBV-CTLs infused	Response in PTLD patients	Complications**	Reference
Gustafsson et al. (2000)	6	rising EBV load after HSCT	HSCT-donor	Expansion	2–4	2 × 10^7^/m² to 4 × 10^7^/m²	NA		[126]
Haque et al. (2002)	8	PTLD after SOT or HSCT	Third-party	Expansion	1–6	1 × 10^6^/kg to 6 × 10^6^/kg	3/8 CR 1/8 PR		[90]
Sun et al. (2002)	2	2 PTLD after SOT	Third-party	Expansion	3	1.5 × 10^7^/kg	2/2 CR		[127]
Comoli et al. (2007)	4	1 rising EBV load 3 PTLD after HSCT	HSCT donor	Expansion	2–4	1.5 × 10^6^/kg to 5.5 × 10^6^/kg	3/3 CR		[119]
Gandhi et al. (2007)	3	PTLD after SOT	Third party	Expansion	2–8	2 × 10^6^/kg to 16 × 10^6^/kg	2/3 CR		[128]
Haque et al. (2007)	33	PTLD after SOT or HSCT	Third-party	Expansion	4	8 × 10^6^/kg	14/33 CR 3/33 PR	*De novo* alloantibodies in 1/33 pts	[89]
Barker et al. (2010)	2	PTLD after CBT	Third-party	Expansion	5-6	5 × 10^6^/kg to 6 × 10^6^/kg	2/2 CR		[129]
Heslop et al. (2010)	114	101 prevention and 13 treatment of PTLD after HSCT	HSCT-donor	Expansion	1–3	2 × 10^7^/m² to 5 × 10^7^/m²	11/13 CR no PTLD in the prevention group	Swelling at side of disease	[130]
Moosmann et al. (2010)	6	PTLD after HSCT	HSCT-donor	Caption	1	0.3 × 10^6^ to 7.4 × 10^6^ (abs.)	3/6 CR		[94]
Uhlin et al. (2012)	1	PTLD after CBT	Haploidentical, third-party donor	Caption	1	1.1 × 10^4^/kg	1/1 CR		[93]
Doubrovina et al. (2012)***	19	PTLD after HSCT	14 HSCT-donor 5 third party	Expansion	3	3 × 10^6^/kg	13/19 CR		[131]
Icheva et al. (2013)	10	2 EBV reactivation 8 PTLD after HSCT	HSCT-donor	Caption	1-2	1.5 × 10^2^/kg to 5.4 × 10^4^/kg	6/8 CR	Skin-GvHD I-II° in 1/10 pts	[95]

*Expansion: EBV-CTLs were expanded *in vitro* using repetitive stimulation with autologous, EBV-transformed B cells (lymphoblastoid cell lines (LCL); Caption: EBV-specific T cells were labeled with magnetic beads and isolated via a magnetic column without *ex vivo* expansion; **restricted to reported CTL-related complications; in regard to GvHD, only *de novo* GvHD is reported; ***restricted to patients receiving manipulated EBV-CTLs.

CBT: cord blood transplantation; CR: complete remission; EBV-CTL: EBV-specific cytotoxic T-lymphocytes; HSCT: hematopoietic stem cell transplantation; GvHD: graft-versus-host disease; NA: not available; PR: partial response; PTLD: posttransplant lymphoproliferative disease; Pts: patients; SOT: solid organ transplantation.
